# Addressing the challenges of reoperative robotic-assisted sacrocolpopexy

**DOI:** 10.1590/S1677-5538.IBJU.2018.0037

**Published:** 2018

**Authors:** Wilson Lin, Nitya Abraham

**Affiliations:** 1 Yeshiva University Albert Einstein College of Medicine, NY, USA; 2 Department of Urology, Montefiore Medical Center, NY, USA

## Abstract

Sacrocolpopexy is the gold-standard repair for apical pelvic organ prolapse (POP). However, over half of women with POP who undergo the surgery experience recurrence, particularly those with higher preoperative stage, younger age, and greater body weight. We address the challenges of repairing recurrent POP in a patient with a prior transabdominal mesh sacrohysteropexy.

## INTRODUCTION

A 50-year-old woman complaining of vaginal pressure presented with Stage II prolapse. She had three previous abdominal surgeries including an open sacrohysteropexy with retropubic sling placement. Given her young age and desire to maintain vaginal length, we opted for robotic supracervical hysterectomy and sacrocolpopexy.

## RESULTS

As a result of the patient's prior surgeries, bowel and bladder were adherent to the uterus and required dissecting off prior to hysterectomy. Mesh was scarred into the peritoneum overlying the uterus and thus left in situ as the uterus was amputated. Due to insufficient peritoneum to cover the new mesh, a flap was created from the anterior abdominal wall. Seven months later, the patient's symptoms had resolved and her POP-Quantification measurements were improved.

## DISCUSSION

Managing recurrent POP after prior sacrocolpopexy is complex due to scarring and concern for secondary repair durability. Repeat robotic mesh colpopexy is an option, but a vaginal approach may be easier. Preoperative cystoscopy, urodynamics, and upper urinary tract imaging should be considered. Intraoperative ureteral stent placement can help identify the right ureter. Cystoscopy should be performed at the end of the surgery to check for bladder or urethral injury. Ultimately, the surgical method should be individualized.

## ARTICLE INFO

**Available at: http://www.intbrazjurol.com.br/video-section/20180037_Lin_et_al**

**Int Braz J Urol. 2018; 44 (Video #20): 1263-4**
